# Reasons for the Practice, Abandonment, and Non-Practice of Extracurricular Physical Activity and Sport Among Primary and Secondary School Students in Cantabria: What Can We Do About It?

**DOI:** 10.3390/sports13020044

**Published:** 2025-02-07

**Authors:** Martín Barcala-Furelos, Iván González-Gutiérrez, Josune Rodríguez-Negro, Marcos Mecías-Calvo, Rubén Navarro-Patón

**Affiliations:** 1 Faculty of Education Sciences, Universidade de Santiago de Compostela, 15782 Santiago de Compostela, Spain; martin.barcala@usc.es; 2 Research Group on Motor Skills, Physical Education, and Health, Universidade de Santiago de Compostela, 27001 Lugo, Spain; ivan.gutierrez@uneatlantico.es (I.G.-G.); ruben.navarro.paton@usc.es (R.N.-P.); 3 Faculty of Social and Human Sciences, Universidad Europea del Atlántico, 39011 Santander, Spain; 4 Faculty of Education, Pontifical University of Salamanca, 37007 Salamanca, Spain; 5 Department of Didactics of Musical, Plastic and Corporal Expression, Faculty of Education, University of the Basque Country (UPV/EHU), 48940 Leioa, Spain; 6 Faculty of Teacher Training, Universidade de Santiago de Compostela, 27001 Lugo, Spain

**Keywords:** physical inactivity, sedentary lifestyle, obesity, leisure, motivation

## Abstract

(1) Background: Physical education at school is not able to meet the need for physical activity and sport (PA and S) established by international organizations, making it necessary to implement its practice outside school hours. This study aimed to find out the reasons for practicing, abandoning, and never having practiced PA and S outside school hours among students of Primary Education (PE) and Secondary Education (SE) in Cantabria (Spain). (2) Overall, 1038 students participated (349 from PE and 689 from SE), consisting of 512 boys and 526 girls between 10 and 17 years old (M = 12.92; SD = 1.92). They completed an ad hoc questionnaire with 21 questions about reasons for practicing (12 items), abandoning (3 items), and never having practiced PA and S (6 items) between the months of May and June 2024. (3) Results: As for active students, boys argue that they do so because of the influence of friends (*p* = 0.024), search for excitement (*p* = 0.002), liking PA and S (*p* = 0.022), and entertainment (*p* = 0.001). In PE, compared to SE, the most important factors are excitement (*p* < 0.001), health (*p* = 0.005), and liking PA and S (*p* = 0.022). Students who abandon PA and S do so because of the competitive environment (*p* = 0.001), with boys predominating. SE students highlight reluctance and laziness (*p* < 0.001) and the loss of liking PA and S (*p* = 0.013). Students who have never practiced PA and S do so because they do not find any sport motivating (*p* = 0.047) and because of reluctance and laziness (*p* = 0.018), especially among girls. In SE, the differences appear due to reluctance and laziness (*p* = 0.009) and because friends do not practice PA and S (*p* = 0.049). (4) Conclusions: Boys prioritize emotional and competency aspects, while girls focus on social aspects and happiness. PE students tend to participate in sports for fun and to improve their skills, while SE students tend to show reluctance and laziness and a loss of interest in PA and S.

## 1. Introduction

The practice of physical activity and sport (PA and S) contributes to maintaining a state of health and physical well-being [1], as well as to cognitive, emotional, and social development [2]. Therefore, especially during the early stages of life [3] and particularly in adolescence, due to behavioral, emotional, and physiological changes [4], the practice of regular physical activity is an essential element for the acquisition of a healthy lifestyle [5]. In this sense, the World Health Organization [6] recommends that children and adolescents between 5 and 17 years of age do some type of physical activity for at least 60 min a day, as well as reduce passive leisure and sedentary behavior. Currently, studies indicate that, regardless of age or gender [7], the recommendations established by the WHO regarding the practice of minimum established physical-sports activity are not met [6,8], since 81% of adolescents do not reach it [9], which directly influences the quality of life, increasing the pathologies related to a sedentary lifestyle [10,11], such as obesity [12].

On the other hand, it is known that the practice of extracurricular PA and S by adolescents produces positive effects on quality of life [13]. However, sedentary behaviors are increasingly common at school age [14]. In this sense, Castillo-Retamal et al. [15] suggest that the school environment is the ideal place to promote physical activity and that the participation of students in properly structured physical activity programs contributes to improving physical condition, cooperation, and social skills and reducing anxiety and depression. Therefore, it is necessary to implement effective strategies to promote healthy lifestyles [11] and promote PA and S as soon as possible [16,17].

The physical education school subject has undergone a substantial evolution in recent years [18], since both the PE curriculum [19] and that of SE [20] seek that students internalize the regular and systematic practice of physical activity and identify all those behaviors that are harmful to health or that negatively affect it. However, the Spanish educational system has a weekly physical education teaching load below the hours recommended by international organizations [21]. Although there are studies showing that short sessions (15 min) of strength training reduce fat mass [22] or that active breaks increase the motor involvement of students during their stay at the educational center [23], it should not be thought that this is adequate and sufficient [18,22], since, although there is an improvement in health in a short period of time, the international recommendations on physical activity are not yet met [6]. Thus, educational centers are not capable on their own of implementing programs that contribute to students’ adherence to physical activity, highlighting the need to promote its practice beyond school physical education hours [7].

In this sense, it is necessary to understand that the reasons for regularly practicing PA and S are influenced by various factors, such as, for example, current social trends [24]; the impact of teachers, friends, and parents from primary to secondary school [25]; and the motivation towards practicing PA and S [26], which may vary depending on gender and age [27].

Among PE students, there is a general trend in favor of boys towards greater motivation to practice PA and S due to aspects related to health, weight and body image, muscular strength and endurance, and competition [28]. However, these results also vary by age, with 10-year-old girls showing greater motivation than 11- and 12-year-olds for health, weight and body image, or social recognition, and 11-year-old boys showing greater motivation for fun and well-being or competition, among others [28].

Friendships are another factor that influences the motivation to practice PA and S, both in PE and SE education [29], and this influence is accentuated with age [30], so that practicing PA and S with friends in adolescence represents a determining factor in regular PA and S practice in SE [24].

In addition, there are more factors that determine the motivation towards the regular practice of PA and S, among which is the search for an optimal state of health [31,32], the improvement of basic physical capacities [33], and aspirations to be professional athletes [34]. Regarding the latter, sports specialization at early ages is becoming more frequent [35], causing in many cases the “burnout” syndrome, being one of the main reasons for abandoning the PA and S practice [36]. Depending on age, this dropout occurs more frequently in SE [27], despite the fact that SE students attach great importance to carrying out extracurricular PA and S activities [37].

On the other hand, the main reasons for abandoning the practice of PA and S are lack of time [38], lack of studies [5], and preference for other leisure activities [17,33,39]. Depending on gender, taking into account the study by [40], carried out on SE students, girls show a higher rate of never having practiced PA and S or having abandoned them, indicating personal reasons, laziness and reluctance, or preferences for other activities as the main causes.

For all the above, knowing the reasons why young people from PE and SE practice, abandon, or never practice PA and D is crucial to improve their adherence [32] and thus generate healthy lifestyle habits [5]. Therefore, the objective of this study was to find out the reasons for practicing and abandoning and for never having practiced extracurricular PA and S among PE and SE students from educational centers in Cantabria, based on gender and educational stage.

## 2. Materials and Methods

### 2.1. Study Design

This selective study was carried out from a non-probabilistic cross-sectional descriptive methodological perspective [41] in a sample of Cantabrian schoolchildren in Primary and Secondary Education.

### 2.2. Participants

The sample selection for this study was non-probabilistic and by convenience, based on the subjects accessed due to the geographical proximity of the researchers, from Primary and Secondary Education centers supported with public funds from the Ministry of Education of Cantabria (Spain). A total of 17 centers were invited to participate (10 Secondary, 6 Primary, and 1 Primary and Secondary), covering a total of 1164 schoolchildren. The inclusion criteria to participate in the research were the following: (a) being enrolled in the courses from 5th of Primary to 4th of Secondary Education; (b) not suffering from a physical or mental health problem that prevented the completion of the questionnaire; (c) presenting the informed consent signed by their legal guardians; and (d) filling out all the questions posed in the questionnaires. Of the 1164 schoolchildren, 126 were excluded for not meeting any of the inclusion criteria. The sample size was calculated using the finite population formula [42], which would require 1032 schoolchildren for a 95% confidence level and a 3% margin of error.

### 2.3. Study Variables

The dependent variables (discrete quantitative variables) considered in this study are the reasons for practicing (12 items), abandoning (3 items), and never having practiced PA and S (6 items). The independent variables (dichotomous categorical qualitative variables) are gender (boys vs. girls) and educational level (primary education vs. secondary education).

### 2.4. Instrument

An ad hoc questionnaire was used, consisting of 4 blocks: Block 1 [general data; 5 items (gender, age, course and stage, and PA and S practice)]; Block 2 (reasons for PA and S practice; 12 items); Block 3 (reasons for abandoning PA and S; 3 items); and Block 4 (reasons for never having practiced PA and S; 6 items). For its development, the Scale for Measuring Motives for Physical Activity [43] and the Questionnaire of Motives for Abandoning and Not Practicing Physical-Sports Activity in Spanish Adolescents [44] were taken into account. The range of possible values for Blocks 2, 3, and 4 was 1–4, with 1 being totally disagree and 4 being totally agree. Depending on whether participants answered yes to question E of the questionnaire, yes before but not now, or I have never practiced PA and S, they responded exclusively to Blocks 2, 3, or 4, respectively, without being able to respond to the other two blocks. A 4-value scale was used to prevent participants from using the mean value to give their answers, which could have been given on a scale of 1 to 3.

### 2.5. Procedures

The principal of each of the 17 schools in Cantabria (Spain) participating in this study was contacted, as well as the physical education teachers, chosen by convenience, who were personally informed by the researchers, who explained the objective of the study to them and requested their permission to carry it out. Once the approval was obtained at the center, an informed consent form was sent to the legal guardians of the schoolchildren, providing them with all the necessary information about the study, highlighting the voluntary nature of participation and withdrawal at any time. After obtaining informed consent, the researchers administered the questionnaire, dedicating 30 min to completing it during the physical education sessions during the third quarter of the 2023–2024 school year, using the same measurement strategies and techniques in all educational centers to avoid information bias. The evaluations were carried out by the same people, always using the same instrument and procedure, providing standardized instructions on how to cover it and also addressing any questions that arose throughout the process.

The research has followed the procedures of the Declaration of Helsinki at all times and was approved by the Ethics Committee of the European University of the Atlantic on 6 May 2022, with the code number CEI21_2022.

### 2.6. Statistical Analysis

For all analyses, the SPSS statistical package (SPSS v.26, IBM Corporation, New York, NY, USA) was used, with a level of statistical significance of *p* < 0.05. Measures of central tendency (mean; standard deviation) were used to express quantitative data, and frequencies and percentages were used to express qualitative data. The internal consistency reliability of the instrument scores was estimated with McDonald’s Omega for each factor [i.e., reasons for practicing (12 items), reasons for abandoning (3 items), and reasons for never having practiced PA and S (6 items)]. A multivariate analysis of variance (MANOVA) was used to determine the possible effect of educational level (primary vs. secondary) and gender (boy vs. girl) on the questionnaire variables [reasons for practicing PA and S (12 items), reasons for abandoning (3 items), and reasons for never having practiced PA and S (6 items)]. The calculation to determine the interaction between both factors was performed using the Bonferroni statistic. The effect size was calculated in terms of eta squared (η^2^), considering the effect size small (0.01), medium (0.06), or large (0.14 or higher).

## 3. Results

A total of 1038 primary (349) and secondary (689) schoolchildren, 526 (50.67%) girls and 512 (49.33%) boys aged between 10 and 17 years (M = 12.92; SD = 1.92), answered the questionnaire questions based on the previous answer given to the PA and S practice question (yes; before yes, now no; never). A total of 779 schoolchildren (75.04%) were practicing PA and S, 209 schoolchildren (20.13%) were no longer practicing PA and S, and 50 schoolchildren (4.81%) had never practiced PA and S. Table 1 shows the reasons for practicing PA and S of schoolchildren according to gender and educational stage.

Likewise, the reliability analysis yielded adequate results for the variables studied (McDonald’s Omega > 0.700). This includes the questions corresponding to participants who practiced PA and S (Omega = 0.767), participants who previously practiced PA and S and now do not do so (Omega = 0.704), and finally, participants who have never practiced PA and S (Omega = 0.764). Table 2 shows the reasons for practicing PA and S of schoolchildren according to gender and educational stage.

The results of the MANOVA in relation to the reasons for practicing PA and S (Table 2), based on the educational stage factor, indicate that there are statistically significant differences in “Because I like risk and excitement” [F (1, 775) = 15.169, *p* < 0.001, η^2^ = 0.019]; “To maintain or improve my health (be healthy)” [F (1, 775) = 8.172, *p* = 0.004, η^2^ = 0.010]; “To be in good shape” [F (1, 775) = 8.170, *p* = 0.004, η^2^ = 0.010]; “To release tension and expend energy” [F (1, 775) = 18.565, *p* < 0.001, η^2^ = 0.023]; “Because I like practice sports” [F (1, 775) = 5.279, *p* = 0.022, η^2^ = 0.007]; “To please my parents” [F (1, 775) = 4.585, *p* = 0.033, η^2^ = 0.006]; “Because I want to be a professional athlete” [F (1, 775) = 22.161, *p* < 0.001, η^2^ = 0.027]; “To make new friends” [F (1, 775) = 14.038, *p* < 0.001, η^2^ = 0.018]; “Because I have fun and have a good time” [F (1, 775) = 10.436, *p* = 0.001, η^2^ = 0.013]; and “To improve my skills” [F (1, 775) = 6.980, *p* = 0.008, η^2^ = 0.009]. In all the variables studied, PE students obtain higher scores than those in SE, except in “To be in good shape”, “To release tension and expend energy”, and “To improve my skills”.

The results of the MANOVA, based on the gender factor, indicate that there are statistically significant differences in “Because my friends also play them” [F (1, 775) = 5.151, *p* = 0.024, η^2^ = 0.007]; “Because I like risk and excitement” [F (1, 775) = 9.684, *p* = 0.002, η^2^ = 0.012]; “To release tension and expend energy” [F (1, 775) = 8.157, *p* = 0.004, η^2^ = 0.010]; “Because I like practice sports” [F (1, 775) = 13.118, *p* < 0.001, η^2^ = 0.016]; “Because I want to be a professional athlete” [F (1, 775) = 60.799, *p* < 0.001, η^2^ = 0.072]; “Because I have fun and have a good time” [F (1, 775) = 5.781, *p* = 0.016, η^2^ = 0.007]; “Because I like to compete” [F (1, 775) = 82.815, *p* < 0.001, η^2^ = 0.095]; and “To improve my skills” [F (1, 775) = 9.791, *p* = 0.002, η^2^ = 0.012]. In these variables, boys score higher than girls except in “To maintain or improve my health (be healthy)”.

Regarding the interaction of both factors, there are statistically significant differences in “Because I like risk and excitement” [F (1, 775) = 6.404, *p* = 0.012, η^2^ = 0.008], where PE boys get higher results than those in SE; and “To be in good shape” [F (1, 775) = 6.338, *p* = 0.010, η^2^ = 0.008], where girls in PE get higher scores than those in SE.

The results of the MANOVA regarding the reasons for abandoning PA and S practice (Table 3), based on the educational stage factor, indicate that there are statistically significant differences in “Due to reluctance and laziness” [F (1, 205) = 19.152, *p* < 0.001, η^2^ = 0.80] and “Because I no longer like playing sports” [F (1, 205) = 6.262, *p* = 0.013, η^2^ = 0.028]. In all the variables studied, SE students obtained higher scores than those in PE. The results of the MANOVA based on the gender factor indicate that there are only statistically significant differences in “Due to the competitive environment” [F (1, 205) = 10.437, *p* = 0.001, η^2^ = 0.046], with higher scores in boys.

The results of the MANOVA on the reasons why they have never practiced PA and S (Table 4), based on the educational stage factor, indicate that there are statistically significant differences in “Due to reluctance and laziness” [F (1, 46) = 7.531, *p* = 0.009, η^2^ = 0.141]; “Because there are no sports facilities nearby” [F (1, 46) = 7.966, *p* = 0.012, η^2^ = 0.133]; and “Because my friends don’t play sports” [F (1, 46) = 4.233, *p* = 0.049, η^2^ = 0.083]. In all the variables studied, SE students give higher scores on the reasons for never having practiced PA and S than PE students.

The results of the MANOVA based on the gender factor show statistically significant differences in “Because I haven’t found any sport that motivates me” [F (1, 46) = 4.161, *p* = 0.047, η^2^ = 0.085]; “Because I’m not having fun, I’m bored” [F (1, 46) = 5.702, *p* = 0.018, η^2^ = 0.119]; and “Because I don’t like physical education at school at all” [F (1, 46) = 6.888, *p* = 0.010, η^2^ = 0.139], with higher scores in girls.

## 4. Discussion

The objective of this research was to find out the reasons for practicing, abandoning, and not having practiced PA and S outside the school environment among PE and SE students from educational centers in Cantabria, based on gender and educational stage.

Regarding the reasons for practicing PA and S, taking gender into account, the results show that there are significant differences, in favor of boys, in aspects related to the liking for physical-sports practice, or the fact of having a good time, staying fit, or improving their skills, as in the study by Pereyra [45]. These results show that fun is one of the most determining elements for the regular practice of physical activity [33]. In the study by Mecías-Calvo et al. [28], the aspects of fun and well-being in PE students do not show significant differences between genders, contrary to what occurs in our study. On the other hand, in the study by Alemany Arrebola et al. [5] on SE students, boys report a liking for health, physical appearance, and fun as some of the main reasons for practicing PA and S, as in our study. Therefore, implementing innovative methodologies in SE where students perceive fun and entertainment could be an effective tool [46], given the tendency towards sedentary behaviors and physical inactivity [14], produced by passive leisure or excessive use of new technologies [47].

A key factor when it comes to practicing PA and S is the influence of teachers, parents, and friends [25]. As students get older, the family and physical education teachers have less influence on them; however, their peers and friends have the opposite effect [30]. In the study by Bennàsser Torrandell et al. [48], it was observed that young people who have friends who play sports and who perceive that their parents help them to play sports do more hours of PA and S per week, as in our study. On the contrary, the lack of PA and S practice by the friends of SE students is an important reason for never having practiced PA and S [48], observing in our study that girls highlight the fact that they do not do PA and S because their friends do not do it either.

Healthy lifestyle habits to improve health, stay fit, or expend energy are some of the determining reasons for regular PA and S practice [31,32]. The results of the present study show that PE students agree more with these reasons than SE students, coinciding with the results of Valero González et al. [49] for PE students and with Romero-Chouza et al. [50] for SE students. Thus, the increase in overweight and obesity, especially in PE compared to SE [51], causes the need to promote the practice of PA and S as soon as possible [16,17]. Regarding the motivation to practice PA and S, based on gender, significant differences are found between boys and girls, with boys providing higher scores in aspects related to improving skills, liking to compete, and expectations of wanting to be a professional. These results agree with those obtained by Santos Labrador and Melero Ventola [34], where boys show greater motivation in aspects related to improving physical condition orientated towards competition.

Regarding the idea of trying to become a professional athlete or the pursuit of sporting performance, the boys in our study have significantly different opinions than the girls, unlike the study by Aznar-Ballesta and Vernetta [40], where no differences were found between boys and girls regarding competitive pressure or stress. In this sense, the competitive environment often demands a high level of commitment and performance that young people, both boys and girls, are not willing to tolerate, becoming a reason for abandoning sports practice [52].

Another reason for abandoning the PA and S practice is the fact that they no longer like to practice sport, either due to reluctance or laziness. These results coincide with the results of the study by Aznar-Ballesta and Vernetta [40], where the preference for other activities or laziness and reluctance are some of the main causes of abandonment in both sexes, although girls show a slightly higher tendency to abandon PA and S practice. Taking into account the educational stage, as in the study by Barcala-Furelos et al. [39], SE students show other preferences in their leisure time that motivate them more. On the other hand, the results obtained by students who have never practiced PA and S, taking gender into account, show that girls indicate that they do not like physical education at school, nor have they found sports that motivate or entertain them. Therefore, girls show less motivation and predisposition towards physical education than boys [53]. This may be due to the existence, still, of masculinizing stereotypes associated with female athletes that cause indifference towards PA and S, opting for other types of activities with a high social component that bring them more happiness [54], which could explain the reason for never having practiced sport or even abandoning it [55].

Lastly, SE students indicate that one of the reasons for never having practiced sport is not having facilities nearby, which highlights the need to apply programs based on active methodologies that offer different alternatives to traditional ones to occupy leisure time and space [56].

Regarding the limitations found in the development of the study, the following were found: (1) For data collection, an ad hoc questionnaire was used, with the limitations that this instrument implies. The employed tool, despite not being validated, has been used in past works [43,44]. Usually, it is answered in a socially expected way, and as such, there may be a lack of sincerity in their responses, as they may have responded dishonestly for fear of being judged or because they wanted to present a more favorable image of themselves; they may have responded without reflecting and analyzing the question itself before answering, or there may have been differences in the understanding and interpretation of the respondents, which may lead to inconsistent responses and thus affect the reliability of the data collected. (2) The research has only been carried out in the Autonomous Community of Cantabria, so it would be interesting to incorporate educational institutions from other communities and also from other countries. (3) No differentiation has been made between public and private centers, or between rural and urban centers. (4) Those centers that have a specific plan for health-promoting schools have not been taken into account to find out whether the students have a greater motivation or predisposition towards the PA and S practice, nor have those centers that promote health through their own institutional plan been taken into account. (5) Those centers that offer extracurricular activities in the afternoon have not been taken into account either.

## 5. Conclusions

This research revealed significant differences in students’ motivation to engage in and abandon PA and S outside of school based on gender and educational stage. The results showed that boys tend to place more importance on aspects such as excitement, competition, and stress relief, while girls place more importance on social interaction and happiness. Furthermore, it was observed that PE students engage in PA and S for fun and to improve their skills, while SE students showed more passivity and reluctance when it came to engaging in PA and S. These findings suggest that gender and educational stage differences should be taken into account when designing physical education programs to foster an inclusive environment that encourages active participation by all students and promotes physical and mental well-being. In summary, understanding these motivations is essential to developing effective strategies that encourage students to practice physical education and development, both in and out of school.

Students who have never played sports give various reasons, with notable differences depending on gender and educational stage. Girls report higher levels of disinterest and boredom, while ES students report reluctance and lack of nearby facilities as important barriers.

## 6. Practical Applications

Based on the research findings, a number of specific interventions and programs are proposed to address differences in students’ motivation towards physical activity and sport (PA and S). (1) Tailored campaigns: Develop awareness and motivation campaigns that highlight the specific benefits of PA and S for each gender and educational stage. For example, for boys, excitement, competition, and stress relief should be emphasized, while for girls, social interaction and happiness should be highlighted. For primary school students, fun and skill enhancement should be promoted, and for secondary school students, passivity and lack of interest should be addressed with more engaging and accessible activities. (2) Extracurricular activity programs: create sports clubs and workshops that cater to the specific interests of each group; for example, this includes competitions for boys, activities such as dance or yoga for girls, and mixed activities that encourage social interaction and cooperation between genders. (3) Inclusive and supportive school policies that promote an inclusive environment for all students: this involves training teachers in motivational techniques and creating an inclusive environment, ensuring that sports facilities are accessible and suitable for all students, and establishing mentoring programs where older or more experienced students can guide and motivate younger ones. (4) Specific interventions. (4.1.) Teachers: To change the motivational profile and generate experiences of satisfaction in class, teachers must address components such as fun, competition, and social relationships. New content and innovative proposals can be developed to avoid routine or boredom, such as alternative sports, new artistic-expressive trends, educational CrossFit, and parkour, among others. In addition, active methodologies must be implemented where students are the true protagonists of the teaching–learning process, using pedagogical models such as cooperative learning, the sports education model, or gamification. (4.2.) Structured learning environments: Provide highly structured learning environments, with appropriate progression, so that students can succeed and progress in their skills. Setting tasks in the form of challenges, providing specific feedback, praising individual improvement, encouraging them, and building confidence will stimulate their motivation to increase their level of motor competence. (4.3.) Positive social relationships: create positive environments based on respect, trust, and empathy, design activities that encourage social interaction among students, and develop proposals that require collaboration, teamwork, and joint resolution of tasks to promote group identity and individual responsibility within the group. (5) Opening up to the community: open up the center and physical education classes to the community, carry out various activities in the immediate surroundings (green spaces, calisthenics park, bike lane, etc.), develop complementary and extracurricular activities in the vicinity of the educational center, using municipal sports facilities, and establish collaboration agreements with sports clubs and schools in the area. These strategies can help to consolidate the practice of PA and S outside the school environment and eliminate barriers or reasons for schoolchildren to abandon PA and S activity.

## Figures and Tables

**Table 1:** Sample of participants by stage, gender, and response.

	Primary Education		Secondary Education		
Answer to Question E of the Questionnaire: Practice Physical Activity and Sport	Boys (n)	Girls (n)	Boys (n)	Girls (n)	Total (n)
Yes (they only answered Block 2)	135	140	290	214	779
Before yes, but now no (they only answered Block 3)	14	44	51	99	209
No, never (they only answered Block 4)	6	9	15	20	50
**Total (n)**	155	193	356	333	1037

**Table 2:** Univariate and multivariate analysis of the reasons for practicing PA and S based on gender and educational stage.

	Primary Education		Secondary Education		Main Effects		Interaction
	Boys (n = 135)	Girls (n = 140)	Boys (n = 290)	Girls (n = 214)	Gender (A) *F*	Stage (B) *F*	A × B *F*
Because my friends also play them (1–4)	1.96 ± 1.02	1.82 ± 0.94	1.94 ± 0.88	1.79 ± 0.81	5.151 *	0.318	0.000
Because I like risk and excitement (1–4)	3.04 ± 0.04	2.63 ± 1.00	2.58 ± 0.98	2.54 ± 0.92	9.684 **	15.169 ***	6.404 **
To release tension and expend energy (1–4)	3.09 ± 0.92	2.83 ± 0.90	3.28 ± 0.76	3.16 ± 0.82	8.157 **	18.565 ***	1.410
Because I like practicing sports (1–4)	3.82 ± 0.51	3.68 ± 0.64	3.74± 0.76	3.56 ± 0.68	13.118 ***	5.279 *	0.198
Because I want to be a professional athlete (1–4)	3.21 ± 1.02	2.74 ± 1.06	2.94 ± 0.98	2.26 ± 1.03	60.799 ***	22.161 ***	1.314
Because I have fun and have a good time (1–4)	3.64 ± 0.68	3.52 ± 0.76	3.48 ± 0.70	3.32 ± 0.78	5.781 *	10.436 **	0.065
Because I like to compete (1–4)	3.34 ± 0.94	2.72 ± 1.06	3.35 ± 0.88	2.64 ± 1.07	82.815 ***	0.169	0.318
To improve my skills (1–4)	3.66 ± 0.57	3.59 ± 0.62	3.04 ± 1.02	3.40 ± 0.69	9.791 **	6.980 **	2.391
To maintain or improve my health (be healthy) (1–4)	3.47 ± 0.70	3.61 ± 0.60	3.41 ± 0.71	3.37 ± 0.71	1.054	8.172 **	2.816
To be in good shape (1–4)	3.57 ± 0.66	3.64 ± 0.50	3.55 ± 0.66	3.37 ± 0.73	1.171	8.170 **	6.638 **
To please my parents (1–4)	1.74 ± 0.96	1.77 ± 1.00	1.67 ± 0.87	1.57 ± 0.71	0.543	4.585 *	0.800
To make new friends (1–4)	2.91 ± 1.01	2.66 ± 0.94	2.52 ± 0.96	2.50 ± 0.99	3.732	14.038 ***	2.713

Note: data are presented as mean ± standard deviation. * *p* < 0.05; ** *p* < 0.01; *** *p* < 0.001.

**Table 3:** Univariate and multivariate analysis of the reasons for abandoning PA and S practice based on gender and educational stage.

	Primary Education		Secondary Education		Main Effects		Interaction
	Boys (n = 15)	Girls (n = 44)	Boys (n = 51)	Girls (n = 99)	Gender (A) *F*	Stage (B) *F*	A × B *F*
Due to the competitive environment (1–4)	2.00 ± 1.13	1.47 ± 0.76	1.74 ± 1.03	1.40 ± 0.58	10.437 **	0.790	0.036
Due to reluctance and laziness (1–4)	1.46 ± 0.51	1.43 ± 0.75	2.03 ± 1.03	2.27 ± 1.06	0.446	19.152 ***	0.494
Because I no longer like playing sports (1–4)	1.26 ± 0.45	1.31 ± 0.63	1.70 ± 0.94	1.51 ± 0.73	0.244	6.262 *	1.064

Note: data are presented as mean ± standard deviation. * *p* < 0.05; ** *p* < 0.01; *** *p* < 0.001.

**Table 4:** Univariate and multivariate analysis of the reasons for never having practiced PA and S based on gender and educational stage.

	Primary Education		Secondary Education		Main Effects		Interaction
	Boys (n = 6)	Girls (n = 9)	Boys (n = 15)	Girls (n = 20)	Gender (A) *F*	Stage (B) *F*	A × B *F*
Because I haven’t found any sport that motivates me (1–4)	1.83 ± 1.17	2.75 ± 0.46	2.07 ± 1.16	2.50 ± 1.05	4.161 *	0.026	0.293
Because I’m not having fun, I’m bored (1–4)	1.00 ± 0.02	1.75 ± 1.03	1.53 ± 0.74	2.25 ± 1.11	5.702 *	3.700	0.004
Because I don’t like physical education at school at all (1–4)	1.00 ± 0.02	1.75 ± 1.16	1.40 ± 0.51	2.25 ± 1.16	6.888 *	2.871	0.089
Due to reluctance and laziness (1–4)	1.50 ± 0.84	1.38 ± 0.51	2.33 ± 1.11	2.40 ± 1.23	0.062	7.531 **	0.062
Because there are no sports facilities nearby (1–4)	1.00 ± 0.02	1.75 ± 0.71	1.53 ± 0.74	1.70 ± 0.86	0.432	7.966 **	1.070
Because my friends don’t play sports (1–4)	1.17 ± 0.40	1.38 ± 0.51	1.53 ± 0.64	2.00 ± 0.97	1.494	4.233 *	0.277

Note: data are presented as mean ± standard deviation. * *p* < 0.05; ** *p* < 0.01.

## Data Availability

The data presented in this study are not available, in accordance with the Regulation (EU) of the European Parliament and of the Council 2016/679 of 27 April 2016 regarding the protection of natural persons with regard to the processing of personal data and the free circulation of these data (RGPD).
